# Randomized clinical trial of ICECaP (Individualized Coordination and Empowerment for Care Partners of Persons with Dementia): Primary mental health and burden outcomes

**DOI:** 10.1371/journal.pone.0309508

**Published:** 2025-01-24

**Authors:** Virginia T. Gallagher, Anna Arp, Ryan Thompson, M. Agustina Rossetti, James Patrie, Shannon E. Reilly, Carol Manning

**Affiliations:** 1 Department of Neurology, University of Virginia, Charlottesville, Virginia, United States of America; 2 Brain Institute, University of Virginia, Charlottesville, Virginia, United States of America; 3 Department of Biostatistics, University of Virginia, Charlottesville, Virginia, United States of America; Institute of Mental Health, SINGAPORE

## Abstract

We examine the efficacy of the Individualized Coordination and Empowerment for Care Partners of Persons with Dementia (ICECaP), an intervention that involves one-on-one individualized support from a dementia care coordinator for a dementia care partner, compared to an active control group. At least once monthly contact is made from a dementia care coordinator to the dementia care partner by telephone, video conferencing, email, or in-person support at clinical visits for the person with dementia. In this pilot randomized unblinded control trial of ICECaP, n = 61 (n = 90 randomized) care partners completed 12-months of the ICECaP intervention and n = 69 (n = 92 randomized) care partners received routine clinical support (controls) in an outpatient memory care clinic at an academic medical center, from which the participants were recruited. Early termination endpoints (death and higher level of care) and trial drop out were comparable across groups. Primary efficacy outcomes were evaluated by comparing changes in care partner mental health, burden, and quality of life from baseline to 12-months between ICECaP and controls. Linear mixed-effects model with covariate adjustment revealed no significant group differences in longitudinal changes on measures of caregiving burden, care partner depression, anxiety, quality of life, or reactions to the behavioral symptoms of the person with dementia. Hypothesized reasons for lack of initial efficacy on primary 12-month outcomes are discussed.

## Introduction

There are over 11 million family members and friends providing unpaid care for persons with dementia in the United States. Informal dementia caregiving is valued at $350 billion and 18.4 billion hours of care per year nationally [1]. Although many dementia care partners experience meaning and fulfillment in the context of their caregiving role, caring for a person with dementia is associated with increased levels of psychosocial stress, depression, and emotional burden [2–9].

Many programs and interventions have been developed to support dementia care partners and improve their mental health, quality of life, and caregiving readiness. Among them, care coordination (also referred to as care navigation) has emerged as a promising, individualized intervention to help care partners and their care recipients with dementia navigate complex health systems, financial/insurance systems, community resources, and the emotional burden of caregiving [10–13]. Traditionally, care coordination involves one trained care coordinator providing support to a one care partner and/or patient with navigation of health systems, resource identification, and some degree of social/emotional support [10–12]. As of 2023, approximately 12 dementia care coordination programs had been developed in the United States [14]. Three programs in the United States have tested care coordination’s impact on care partner burden and mental health using randomized clinical trial (RCT) design [11,15–17]. These programs, in which care coordination was delivered exclusively via telephone, reported improvement in care partner symptoms of depression and caregiving burden after variable duration of the intervention, ranging from 3 to 18 months [11,15–17]. In July 2024, the Centers for Medicare and Medicaid Services launched the Guiding an Improved Dementia Experience (GUIDE) Model, which will fund 390 teams across the country to provide coordinated dementia care. Teams funded by GUIDE are required to provide a care coordinator (referred to as a care navigator in GUIDE) to care partner-dementia patient dyads. As such, it is imperative to understand the efficacy of care coordinator programs in improving the mental health and quality of life dementia care partners.

**Fig 1:**
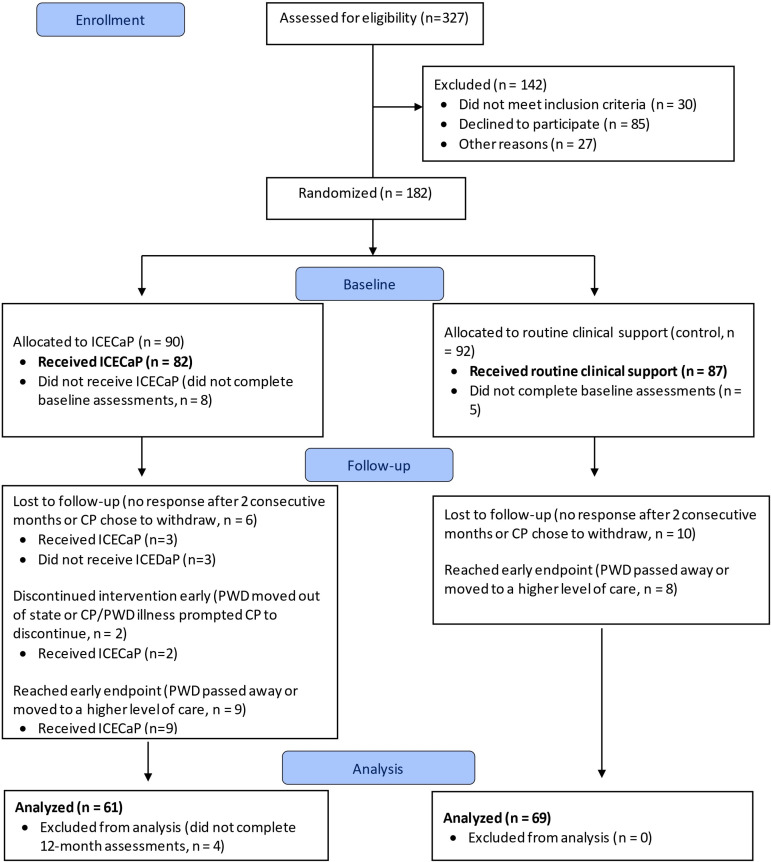
Sample consort flow diagram.

**Fig 2:**
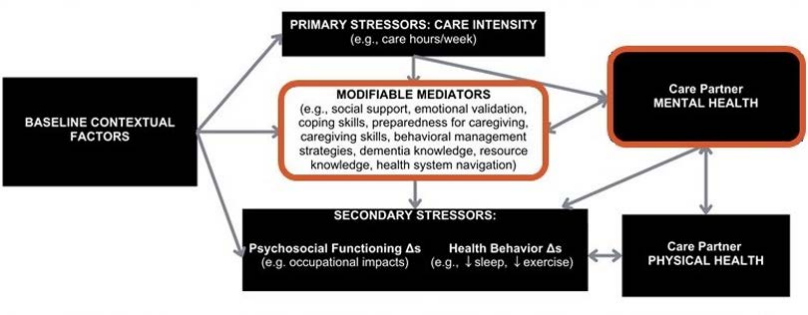
Care partner stress framework.

In 2018, a team at the University of Virginia, along with its partners, developed an intervention for dementia care partners called ICECaP: Individualized Coordination and Empowerment for Care Partners of Persons with Dementia. ICECaP involves individualized elements of care coordination, supportive counseling, psychoeducation, and skills training and is delivered in a hybrid setting—combining an optional initial home visit; ongoing, at least-monthly telehealth interactions via phone, email, and HIPAA-compliant video calls; and accompaniment to clinic visits for the person with dementia. As outlined in the ICECaP protocol paper [18], this intervention was developed based off of the theoretical care partner stress framework (Fig 1), which was adapted from Zarit & Savla’s (2015) biopsychosocial stress process model; this model was updated to incorporate aspects of the Lee (2021) stress process model to easily visualize pathways that can prevent adverse mental and physical health outcomes, as well as Gleason’s model (2022) of the National Institute on Aging (NIA) Health Disparities Research Framework in Alzheimer’s disease risk, in order to better conceptualize social determinants of health [7,19,20]. As depicted in Fig 2, ICECaP aims to improve care partner mental health by targeting and intervening on modifiable mediators (e.g., dementia knowledge, caregiving preparedness, resource knowledge, and social support).

Relative to previous RCTs testing care coordination interventions that focus on care partner mental health [11,15–17], ICECaP is unique in that delivery of care coordination is done in a hybrid environment (contacts between the care coordinator and the care partner occur both in the memory clinic and outside of the clinic, and via face-to-face interaction, email, phone calls, text messaging, and video calls). This study is also novel in its geographic setting (central Virginia), given that previous RCTs of care coordination programs aimed at improving care partner mental health have been conducted in California, Iowa, and Pennsylvania [11,15–17].

Here, we report preliminary, primary efficacy outcomes for a pilot, unblinded randomized control trial (RCT) of ICECaP. Please see Gallagher et al. 2024 for program details, qualification and training of dementia care coordinators, the ICECaP protocol, and analytical plan [18]. We evaluate Aim 1 of the ICECaP RCT: determine whether ICECaP improves care partner burden, symptoms of depression, reaction to the behavioral symptoms of dementia, and quality of life. We hypothesized that after controlling for baseline characteristics, including level of care-recipient functional dependence, care partners in the ICECaP group would significantly improve from baseline to 12 months (post-intervention) on mental health and quality of life measures, whereas controls would not improve.

## Methods

This study was approved by the University of Virginia Institution Research Board for Health Sciences Research and registered with clinicaltrials.gov (NCT04495686). All care partners and persons with dementia underwent a written informed consent process one-on-one with a trained clinical research coordinator (CRC) prior to initiating study procedures. Consent was documented with the signature of the participant and the CRC. All participants were provided with a signed copy of the consent document. This research was supported by the Department of Defense, Virginia Department for Aging and Rehabilitate Services, and the National Institutes of Health.

### Recruitment

As reported in the published protocol [18] and the feasibility and acceptability data for the ICECaP clinical trial [21], persons with dementia and their care partners were recruited from March 1, 2021 to September 30, 2022 from the University of Virginia’s multidisciplinary Memory and Aging Care Clinic (MACC). Care partners were required to be aged ≥18 years, possess basic spoken and written English skills, have home-based internet access, and self-identify as the primary care partner for a patient diagnosed with mild to moderate dementia who was living in the community (e.g., not in a continuing care facility). During the course of the trial, eligibility criteria was expanded to include patient populations being overlooked for recruitment. Changes made included lowering age requirements, and including multiple etiologies of dementia. Care partners were excluded if they were aged <18 years, or if their associated care recipient with dementia resided in an assisted living facility or nursing home and/or had more than one follow-up multidisciplinary appointment in MACC following their dementia diagnosis. Using a random permuted block randomization scheme generated by the study statistician and implemented by the clinical research coordinator at enrollment, care partners were randomly assigned to 12-months of ICECaP or 12-months of routine clinical care (controls) using PROC plan procedure of SAS version 9.4 (SAS Institute Inc., Cary, NC). Fourteen blocks of ten assignments were generated: five ICECaP and five active control assignments, in random order. This study was unblinded.

### Target sample size

Based on an a priori power analysis, the target sample size was n = 140 CPs, (50% ICECaP and 50% control) to achieve at least 0.80 power for detecting a small-to-medium Cohen’s *d* effect size (*d* =  0.3 to 0.5) when comparing baseline to 12-month mean change in the care partner’s depression score (Center for Epidemiological Studies Depression Scale–Revised) between ICECaP and controls and when comparing the baseline to 12-month mean change the care partner’s burden score (Zarit Burden Interview) between ICECaP and control groups at a significance level of α = .05 for each outcome measure. Power calculations assumed a 50% ICECaP and 50% control allocation ratio and a 2-sided α = 0.05 significance level null hypothesis test. POWER procedure software of SAS version 9.4 (SAS Institute Inc., Cary NC) was used to conduct the power analysis.

### Sample and attrition

As described in Thompson et al. (under review) [21], of the n = 169 (control n = 87, ICECap n = 82) care partners recruited into the RCT who completed baseline assessments (out of 182 participants randomized), 23.08% were withdrawn from the final sample because within 12-months, the person with dementia died (n = 9), moved to a higher level of care (n = 8), or moved out of state (n = 1); or the care partner chose to withdraw (n = 4), was lost to follow-up despite two attempts to contact (n = 13), or did not complete 12-month assessments (n = 4). See Fig 1. The final sample of care partners for 12-month outcome analyses consisted of n = 69 controls and n = 61 ICECaP who completed baseline and 12-month assessments.

### Procedures and intervention

Please see Gallagher et al. [18] and S1 File for details. Questionnaires listed in Table 1 were completed online by both ICECaP and control groups at baseline and 12-months after baseline. Additional questionnaires for sample characterization included the Katz Index of Independence in ADLs (Katz Basic ADLs) [22], the Lawton-Brody Instrumental ADL Scale (Lawton-Brody Complex ADLs) [23], and the Neuropsychiatric Inventory Questionnaire, all completed by the care partner about the person with dementia [24]. All measures were collected and stored using REDCap [25], hosted by the University of Virginia, and were monitored by the clinical research coordinator for missing data.

**Table 1:** ICECaP Randomized clinical trial primary outcome measures.

Construct	Self-report measure	Measure details
Care partner burden	Zarit Burden Interview (ZBI) [26]	12-item measure assessing degree of caregiving burden
Care partner depression	Center for Epidemiologic Studies Depression Scale—Revised (CESD-R) [27]	20-item measure assessing symptoms of depression
Care partner anxiety	Geriatric Anxiety Inventory (GAI) [28]	20-item measure assessing symptoms of anxiety
Care partner reaction to behavioral symptoms of dementia	Revised Memory and Behavior Problem Checklist (RMBPC) [29]	24-item measure assessing care partner-reported problematic behaviors in the person with dementia and the care partner’s reaction to the behaviors
Care partner quality of life	WHO (Five) Well-Being Index (WHO-5) [30]	5-item measure assessing dimensions of psychological well-being

#### Intervention.

After baseline questionnaires were completed, a trained dementia care coordinator (referred to going forward as care coordinator) called or e-mailed care partners in the ICECap group to schedule the initial contact. To assess each care partner’s needs, care coordinators reviewed care partner responses to questionnaires listed in Table 1, as well as the care partner’s response to two questions assessing topics of interest/need for the care partner (see S2 File). A care coordinator contacted the care partner at least once per month via email, telehealth phone/video, or an in-person meeting for at least 15 minutes. During these contacts, the care coordinator provided supportive counseling and related services based on the care partner’s expressed needs (e.g., behavioral management, safety strategies, case management, healthcare referrals, psychoeducation). Care partners were encouraged to contact care coordinators as needed. After the initial contact, care coordinators were also required to attend regular MACC follow-up appointments with the care partner and their associated person with dementia. These follow-up appointments typically occur every 6 to 12 months.

#### Control group.

Care partners in the control group received standard care as provided by the MACC (e.g., follow-up appointments every 6 to 12 months). They filled out the same questionnaires at baseline and 12 months as the care partners in the ICECaP intervention group.

### Statistical analysis

#### Data summarization.

Descriptive categorical data were summarized by frequencies (n) and relative frequencies (%), and descriptive continuous scaled data were summarized by either the mean and standard deviation (SD) or the median and interquartile range of the distribution.

#### Baseline analysis.

Although randomization imbalance with respect to caregiving characteristics of the ICECaP and control groups would be rare, sensitivity analyses were conducted to assess for such imbalances. Statistical comparisons of distribution means were conducted via the Welch two-sample t-test, while statistical comparisons of distribution medians were conducted by the Wilcoxon two-sample Rank Sum test. Statistical comparisons of the relative frequencies of each characteristic were conducted by the Pearson two-sample exact test.

#### Efficacy analyses.

Statistical analyses were conducted using the statistical software package SAS version 9.4 (SAS Institute Inc., Cary NC). Pre- to post-intervention 12-month changes in care partner burden (ZBI total score), depression (CESD-R total score), anxiety (GAI total score), reaction to dementia symptoms (RMBPC Reaction score) and quality of life (WHO-5 score) were the focus of the ICECaP trial efficacy analyses. Each efficacy analysis was conducted using a two-step analytical approach. In step 1, a linear mixed-effects model was used to estimate, the intervention-specific mean pre- to post-intervention 12-month efficacy outcome change, under the relaxed heterogenous versus homogenous equal variance assumption. A linear mixed model-derived one-sample t-test was then used to test the null hypothesis that the mean pre- to post-intervention 12-month efficacy outcome change is equal to zero (versus the alternative: not equal to zero). A two-sided α = 0.05 significance level was used. In step 2, a linear mixed-effects model with covariate adjustment was used to test the null hypothesis that the mean efficacy outcome change from baseline to 12-months is the same for ICECaP and controls, after adjusting for baseline Katz Index of Independence in (Basic) ADLs and Lawton-Brody Instrumental (Complex) ADL Scale scores. A two-sided α = 0.05 significance level was used as rejection rule for this null hypothesis test. Analysis was based on the intention-to-treat (ITT) principle.

## Results

### Baseline results

Demographic and caregiving characteristics are presented in Table 2. There were no missing data in the demographics or outcomes variables for this study.

**Table 2:** Care partner demographic and caregiving characteristics.

	ICECaP (n = 61)	Controls (n = 69)
Years of age [M(SD)]	64.8 (12.3)	64.9 (11.6)
Gender		
Woman	42 (68.9%)	52 (75.4%)
Man	19 (31.1%)	17 (24.6%)
Race/Ethnicity		
Non-Hispanic White	57 (93.4%)	60 (87.0%)
Non-Hispanic Black/African American	3 (4.9%)	4 (5.8%)
Hispanic/Latino	1 (1.6%)	3 (4.3%)
Non-Hispanic American Indian	0 (0.0%)	1 (1.4%)
Highest level of education completed		
High school diploma or GED	5 (8.2%)	5 (7.2%)
Some college	10 (16.4%)	8 (11.6%)
Associate’s degree	4 (6.6%)	8 (11.6%)
Bachelor’s degree	27 (44.3%)	25 (36.2%)
Master’s degree	11 (18.0%)	15 (21.7%)
Doctoral degree	4 (6.6%)	6 (8.7%)
Employment status		
Retired	24 (39.3%)	39 (56.5%)
Full-time	15 (24.6%)	15 (21.7%)
Part-time	9 (14.8%)	2 (2.9%)
Unemployed	2 (3.3%)	3 (4.3%)
Monthly income (USD)		
1–4,999	24 (39.3%)	19 (27.5%)
5,000–9,999	13 (21.3%)	18 (26.1%)
10,000–14,999	4 (6.6%)	5 (7.2%)
15,000–24,999	4 (6.6%)	11 (15.9%)
Relationship to PWD		
Spouse/Partner	44 (72.1%)	49 (71.0%)
Adult Child	14 (23.0%)	16 (23.2%)
Co-dwelling with PWD		
Co-dwelling	47 (77.0%)	54 (78.3%)
Non-co-dwelling	14 (23.0%)	15 (21.7%)
**Baseline caregiving characteristics**		
CP hours/month supporting basic ADLs for PWD [MD [IQR]]	3.8 [0, 30.0]	0 [0, 48.7]
CP hours/month supporting complex ADLs for PWD [MD [IRQ]]	30.0 [7.1, 9.0]	60.0 [19.0, 120.0]
Katz Index of Independence in (Basic) ADLs for PWD [M(SD)]	1.3 (0.9)	1.6 (1.1)
Lawton-Brody Instrumental (Complex) ADL Scale for PWD [M(SD)]	3.9 (2.2)	3.7 (2.1)
Neuropsychiatric Inventory Questionnaire- PWD Severity Score [M(SD)] [24]	6.1 (5.1)	5.7 (4.7)

Data presented as n (% of group) unless otherwise noted. ADLs,  activities of daily living; CP,  care partner; M(SD),  Mean (Standard Deviation); MD [IQR],  Median [Interquartile Range]; PWD,  person with dementia.

As expected with randomization procedures, there were no significant differences in baseline scores on any primary outcome measures between groups (see Table 3). Within both groups at baseline, average caregiver burden was in the clinically significant range based on suggested ≥11 cut-score from Bedard [31] and ≥13 cut-score from Brazilian community-based data [32]; however, caregiver burden was on average below clinically significant range based on suggested ≥19 in Singapore community-based data [33]. Average depression scores and anxiety scores were below the threshold for at-risk clinical depression (cut-score ≥16 [27]) and anxiety (cut-score ≥9 [28]), respectively. Participants on average endorsed being between “a little” to “moderately” bothered by the person with dementia’s behavioral symptoms; there is no defined clinically significant cutoff in the literature. There is no established cutoff for the WHO-5 quality of life score; however, Topp et al. [30] suggests ≤50% adjusted score (raw score multiplied × 4) indicates poor well-being; adjusted WHO-5 average raw score was 56.84% for ICECaP and 55.08% for controls. Baseline scores on the Lawton IADL were similar to other prior studies with dementia patients (range =  2.8–4.3 [34,35]), and baseline scores on the Katz ADL indicated a severe level of impairment on average [36].

**Table 3:** ICECaP intervention primary 12-month efficacy results.

	ICECaP (n = 61)				Controls (n = 69)				Mixed Models
Construct	Baseline Mean (SD)	12-Month Mean (SD)	Mean ∆ [95% CI]	*P-value*^*1*^ {Cohen’s d}^1^	Baseline Mean (SD)	12-Month Mean (SD)	Mean ∆ [95% CI]	*P-value*^*2*^ {Cohen’s d}^2^	*P-value*^*3*^ {Cohen’s d}^3^
Burden	17.30 (7.84)	18.38 (7.82)	1.25 [−0.56, 3.06]	*0.173* {0.36}	17.74 (7.96)	18.74 (7.58)	1.00 [−0.44, 2.44]	*0.170* {0.34}	*0.955* {0.01}
Depression	9.11 (11.06)	10.43 (10.83)	1.33 [−1.55, 4.21]	*0.358* {0.24}	11.22 (11.15)	13.14 (13.68)	1.93 [−0.10, 3.96]	*0.062* {0.46}	*0.563* {0.11}
Anxiety	4.46 (4.70)	4.31 (4.61)	−0.15 [−1.28, 0.99]	*0.796* {0.07}	4.62 (4.81)	5.01 (4.64)	0.39 [−0.31, 1.10]	*0.272* {0.27}	*0.396* {0.17}
Reaction to dementia sx	1.31 (0.69)	1.25 (0.71)	−0.05 [−0.26, 0.16]	*0.621* {0.13}	1.34 (0.65)	1.40 (0.65)	0.06 [−0.09, 0.21]	*0.441* {0.19}	*0.204* {0.25}
Quality of life	14.21 (5.13)	13.95 (5.31)	−0.40 [−1.62, 0.82]	*0.515* {0.17}	13.77 (4.71)	13.41 (5.20)	−0.38 [−1.41, 0.65]	0.468 {0.18}	*0.954* {0.12}

Constructs were measured using the following questionnaires: Burden =  ZBI-SF (12-item); Depression =  CESD-R; Anxiety =  GAI; Reaction to Dementia sx (Symptoms) =  RMBPC Reaction score; Quality of Life =  WHO-5. *P-values*^1,2^ were derived from linear mixed model one-sample t-test for testing the null hypothesis that the mean change (Δ) from baseline to 12-months is equal to zero. *P-values*^3^ were derived from the linear mixed model two-sample test for comparing the mean change from baseline to 12-months (Δ) between ICECaP and control groups after adjusting for baseline Katz Index of Independence in (Basic) ADLs and Lawton-Brody Instrumental (Complex) ADL Scale for PWD. Cohen’s d is an effect size index used to indicate the standardized difference between two means.

{Cohen’s d}^1,2^ are the Cohen’s d effect-size index values for ICECaP and Control within-group mean 12-month changes (Δ), respectively, and {Cohen’s d}^3^ is the Cohen’s d effect size index value for the covariate adjusted between-group difference in the mean 12-month change (Δ). Analysis was based on the intention to treat principle.

### Longitudinal results

Feasibility and acceptability data are described by Thompson et al. [21]. To briefly review engagement metrics, care partners in the ICECaP group who completed the intervention had an average of 2.2 contacts per month with their assigned care partner, most commonly via email. Care coordinators spent an average of 34.4 minutes per month, per care partner, in direct contact and 33.7 minutes per month, per care partner, conducting indirect activities (e.g., coordinating care without the care partner involved, investigating local resource options, etc.).

Longitudinal outcomes analyses revealed no significant differences in baseline to 12-month change on primary outcome measures between ICECaP and controls (see Table 3). Results were consistent when the baseline Katz Basic ADLs and Lawton-Brody Complex ADLs were included as covariates in analyses.

### Post-hoc analyses

Post-hoc analyses revealed that care partners in the ICECaP group who did not complete the intervention (non-completers, n = 21) endorsed higher baseline scores on the CESD-R compared to care partners in the ICECaP group who completed 12 months of the intervention (completers, n = 61) (*M*_*diff*_ =  −6.31, *t* =  −2.123, *p* = .037). No group differences were observed between non-completers and completers on the Zarit-SF, GAI, RMBPC, or WHO-5 at baseline (*p*s ≥ .173).

Additionally, a post-hoc correlation matrix was conducted to explore the relationships among a) baseline to 12-month change on primary outcome measures and b) ICECaP engagement metrics within the ICECaP group, including total number of care partner-dementia care coordinator contacts (across all modalities combined and separately by in-person, video, phone, email, and snail mail), average contacts per month, direct minutes (total and average per month), and indirect minutes (total and average per month) of care coordinator time. There were no significant correlations after applying a False Discovery Rate of 0.05 using the Benjamini-Hochberg procedure (see S1 Table) [37]. Uncorrected significant correlations between engagement metrics and longitudinal change on outcome measures suggested that increased number of total in-person contacts and total phone contacts across the 12-month intervention was associated with increased caregiver burden from baseline to 12-months (*p*s ≤ .016).

### Harms

There were no harms associated with the intervention. Inherent harms of providing care for a person with dementia include possible depressive episodes.

## Discussion

This paper presents the preliminary, 12-month efficacy results of a pilot RCT of the ICECaP intervention for care partners of persons with dementia. On average, the 12-month intervention did not appear to significantly change care partners’ self-reported levels of burden, depression, anxiety, quality of life, or reaction to behavioral symptoms in the person with dementia. There were no significant differences between the ICECaP intervention group and the control group in key outcomes at baseline, 12-months, or in change from baseline to 12-months. Further, among the ICECaP group, total care coordinator-care partner contacts or average number of contacts per month did not significantly impact the magnitude of change from baseline to 12-months on outcome measures.

There are several possible explanations why there were no significant mental health, burden, or quality of life benefits detected in this 12-month RCT of ICECaP. First, this cohort of dementia care partners did not indicate experiencing elevated mental health distress, severe caregiving burden, or poor quality of life at baseline according to self-report measures. This is consistent with the relatively high socioeconomic status of this sample, who are majority non-Hispanic White and well educated, rendering them less vulnerable to adverse impacts of caregiving. Therefore, a floor effect may be present in which care partners’ self-report measure scores did not have ample room to allow for change in a positive (i.e., improved) direction. Additionally, the relatively high socioeconomic status of the current sample limits generalizability of these findings to care partners from more diverse socioeconomic backgrounds. In sum, future efficacy testing of ICECaP should have more restrictive inclusion criteria to include only care partners who have elevated levels of the target factor (e.g., burden, depression, and/or anxiety) and greater consideration of diverse socioeconomic statuses.

Additionally, no differential group-by-time effects may have been detected in this RCT due to the nature of the control group in this study. Specifically, all care partners included in both the intervention and control group received follow-up care in the UVA Memory and Aging Care Clinic (MACC). Follow-up care in MACC typically involves a one-hour appointment for the person with dementia and their care partner(s) one to two times per year with a multidisciplinary team of neuropsychologists, neuropsychology postdoctoral fellows, a nurse practitioner, a pharmacist, an occupational therapist, and a speech-language pathologist, among other specialties. While these appointments are scheduled for the person with dementia, the accompanying care partner receives information about pharmacological and non-pharmacological behavioral management strategies; long-term care planning support; dementia psychoeducation; psychotherapy and support group options for care partners; information regarding adult day, continuing care, and respite facilities; and other local resources. Further, care partners are often provided emotional support, validation, and encouragement; at times, the care partner and the person with dementia are separated during the appointment so a care partner can receive one-on-one validation and support (e.g., while the person with dementia is completing brief testing to inform treatment recommendations). Although once to twice yearly support in the multidisciplinary clinic is a lower frequency and intensity of support relative to the monthly support care partners receive in ICECaP, it is possible that there is too much overlap in support that was provided to both the ICECaP group and the control group to detect a signal. In the future, it may be more appropriate to restrict the control group to care partners in the community who are on the waitlist for or do not have access to specialty care services.

Lastly, as shown in post-hoc analyses, care partners in the ICECaP group who were withdrawn from the study prior to completion of the intervention reported higher rates of depression at baseline compared to care partners who completed the intervention completed the study. Consequently, this loss of participants who may have benefited from receiving 12 months of ICECaP could have contributed, at least in part, to the lack of a group-by-time effect. Future studies on dementia care partners may wish to include additional assessments at shorter time intervals to capture greater detail on the impacts of study participation and to allow for greater preservation of participant data given the progressive and terminal nature of dementia.

## Supporting information

S1 File Published ICECaP clinical trial protocol. (PDF)

S2 File Future needs assessment. (PDF)

S1 Table Correlation matrix of engagement metrics and outcome data within the ICECaP group. (XLSX)

## References

[1] Alzheimer’s Association. 2024 Alzheimer’s disease facts and figures.

[2] Mahoney R, Regan C, Katona C, Livingston G. Anxiety and depression in family caregivers of people with Alzheimer disease: the LASER-AD study. Am J Geriatr Psychiatry. 2005;13(9):795–801. doi: 10.1176/appi.ajgp.13.9.795 16166409

[3] Abdollahpour I, Nedjat S, Salimi Y, Noroozian M, Majdzadeh R. Which variable is the strongest adjusted predictor of quality of life in caregivers of patients with dementia? Psychogeriatrics. 2015;15(1):51–7. doi: 10.1111/psyg.12094 25515404

[4] Huang S-S. Depression among caregivers of patients with dementia: associative factors and management approaches. World J Psychiatry. 2022;12(1):59–76. doi: 10.5498/wjp.v12.i1.59 35111579 PMC8783169

[5] Sallim AB, Sayampanathan AA, Cuttilan A, Ho R. Prevalence of mental health disorders among caregivers of patients with Alzheimer disease. J Am Med Dir Assoc. 2015;16(12):1034–41. doi: 10.1016/j.jamda.2015.09.007 26593303

[6] Chiao C-Y, Wu H-S, Hsiao C-Y. Caregiver burden for informal caregivers of patients with dementia: a systematic review. Int Nurs Rev. 2015;62(3):340–50. doi: 10.1111/inr.12194 26058542

[7] Zarit SH, Savla J. Caregivers and Stress. In: Fink G, editor. Stress: concepts, cognition, emotion, and behavior. San Diego: Academic Press; 2016. p. 339–44.

[8] Collins RN, Kishita N. Prevalence of depression and burden among informal care-givers of people with dementia: a meta-analysis. Ageing Soc. 2019;40(11):2355–92. doi: 10.1017/s0144686x19000527

[9] Steinsheim G, Malmedal W, Follestad T, Olsen B, Saga S. Factors associated with subjective burden among informal caregivers of home-dwelling people with dementia: a cross-sectional study. BMC Geriatr. 2023;23(1):644. doi: 10.1186/s12877-023-04358-3 37817101 PMC10565959

[10] Bernstein A, Harrison KL, Dulaney S, Merrilees J, Bowhay A, Heunis J, et al. The role of care navigators working with people with dementia and their caregivers. J Alzheimers Dis. 2019;71(1):45–55. doi: 10.3233/JAD-180957 31322558 PMC7004209

[11] Possin KL, Merrilees JJ, Dulaney S, Bonasera SJ, Chiong W, Lee K, et al. Effect of collaborative dementia care via telephone and internet on quality of life, caregiver well-being, and health care use: the care ecosystem Randomized clinical trial. JAMA Intern Med. 2019;179(12):1658–67. doi: 10.1001/jamainternmed.2019.4101 31566651 PMC6777227

[12] Kallmyer BA, Bass D, Baumgart M, Callahan CM, Dulaney S, Evertson LC, et al. Dementia care navigation: building toward a common definition, key principles, and outcomes. Alzheimers Dement (N Y). 2023;9(3):e12408. doi: 10.1002/trc2.12408 37533688 PMC10392594

[13] Hirschman KB, McHugh M, Morgan B. An integrative review of measures of transitions and care coordination for persons living with dementia and their caregivers. Alzheimers Dement (N Y). 2023;9(3):e12391. doi: 10.1002/trc2.12391 37555017 PMC10404587

[14] Anthonisen G, Luke A, MacNeill L, MacNeill AL, Goudreau A, Doucet S. Patient navigation programs for people with dementia, their caregivers, and members of the care team: a scoping review. JBI Evid Synth. 2023;21(2):281–325. doi: 10.11124/JBIES-22-00024 36449660 PMC10578521

[15] Chu P, Edwards J, Levin R, Thomson J. The use of clinical case management for early stage Alzheimer’ patients and their families. Am J Alzheimers Dis Other Demen. 2000;15(5):284–90. doi: 10.1177/153331750001500506

[16] Mavandadi S, Wray LO, DiFilippo S, Streim J, Oslin D. Evaluation of a telephone-delivered, community-based collaborative care management program for caregivers of older adults with Dementia. Am J Geriatr Psychiatry. 2017;25(9):1019–28. doi: 10.1016/j.jagp.2017.03.015 28433550

[17] Kokorelias KM, Shiers-Hanley JE, Li Z, Hitzig SL. A systematic review on navigation programs for persons living with dementia and their caregivers. Gerontologist. 2023;63(8):1341–50. doi: 10.1093/geront/gnac054 35439813

[18] Gallagher VT, Reilly SE, Worthington G, Patrie J, Manning C. Individualized Coordination and Empowerment for Care Partners of Persons with Dementia (ICECaP): study rationale and protocol. Contemp Clin Trials. 2024;137:107418. doi: 10.1016/j.cct.2023.107418 38135211

[19] Lee K, Yefimova M, Puga F, Pickering CE. gender differences in caregiver burden among family caregivers of persons with Dementia. J Gerontol Nurs. 2021;47(7):33–42. doi: 10.3928/00989134-20210610-03 34191655

[20] Gleason CE, Zuelsdorff M, Gooding DC, Kind AJH, Johnson AL, James TT, et al. Alzheimer’s disease biomarkers in black and non-hispanic white cohorts: a contextualized review of the evidence. Alzheimers Dement. 2022;18(8):1545–64. doi: 10.1002/alz.12511 34870885 PMC9543531

[21] Thompson RC, Gallagher VT, Reilly SE, Arp AM, Manning C. Individualized Coordination and Empowerment for Care Partners of Persons with Dementia (ICECaP): Feasibility and Acceptability. Rochester (NY): Social Science Research Network; 2024. 10.1016/j.cct.2024.107770 PMC11934102 39631536

[22] KATZ S, FORD AB, MOSKOWITZ RW, JACKSON BA, JAFFE MW. Studies of illness in the aged. The index of ADL: a standardized measure of biological and psychosocial function. JAMA. 1963;185:914–9. doi: 10.1001/jama.1963.03060120024016 14044222

[23] Lawton MP, Brody EM. Assessment of older people: self-maintaining and instrumental activities of daily living. Gerontologist. 1969;9(3):179–86. doi: 10.1093/geront/9.3_part_1.179 5349366

[24] Kaufer DI, Cummings JL, Ketchel P, Smith V, MacMillan A, Shelley T, et al. Validation of the NPI-Q, a brief clinical form of the Neuropsychiatric Inventory. J Neuropsychiatry Clin Neurosci. 2000;12(2):233–9. doi: 10.1176/jnp.12.2.233 11001602

[25] Harris PA, Taylor R, Minor BL, Elliott V, Fernandez M, O’Neal L, et al. The REDCap consortium: building an international community of software platform partners. J Biomed Inform. 2019;95:103208. doi: 10.1016/j.jbi.2019.103208 31078660 PMC7254481

[26] Bédard M, Molloy DW, Squire L, Dubois S, Lever JA, O’Donnell M. The Zarit Burden Interview: a new short version and screening version. Gerontologist. 2001;41(5):652–7. doi: 10.1093/geront/41.5.652 11574710

[27] Eaton WW, Smith C, Ybarra M, Muntaner C, Tien A. Center for epidemiologic studies depression scale: review and revision (CESD and CESD-R). The use of psychological testing for treatment planning and outcomes assessment: Instruments for adults, Volume 3, 3rd ed. Mahwah, NJ, US: Lawrence Erlbaum Associates Publishers; 2004. p. 363–77.

[28] Johnco C, Knight A, Tadic D, Wuthrich VM. Psychometric properties of the Geriatric Anxiety Inventory (GAI) and its short-form (GAI-SF) in a clinical and non-clinical sample of older adults. Int Psychogeriatr. 2015;27(7):1089–97. doi: 10.1017/S1041610214001586 25111285 PMC4501012

[29] Teri L, Truax P, Logsdon R, Uomoto J, Zarit S, Vitaliano PP. Assessment of behavioral problems in dementia: the revised memory and behavior problems checklist. Psychol Aging. 1992;7(4):622–31. doi: 10.1037//0882-7974.7.4.622 1466831

[30] Topp CW, Østergaard SD, Søndergaard S, Bech P. The WHO-5 well-being index: a systematic review of the literature. Psychother Psychosom. 2015;84(3):167–76. doi: 10.1159/000376585 25831962

[31] Bédard M, Molloy DW, Squire L, Dubois S, Lever JA, O’Donnell M. The Zarit Burden Interview: a new short version and screening version. Gerontologist. 2001;41(5):652–7. doi: 10.1093/geront/41.5.652 11574710

[32] Gratão ACM, Brigola AG, Ottaviani AC, Luchesi BM, Souza ÉN, Rossetti ES, et al. Brief version of Zarit Burden Interview (ZBI) for burden assessment in older caregivers. Dement Neuropsychol. 2019;13(1):122–9. doi: 10.1590/1980-57642018dn13-010015 31073389 PMC6497029

[33] Yu J, Yap P, Liew TM. The optimal short version of the Zarit Burden Interview for dementia caregivers: diagnostic utility and externally validated cutoffs. Aging Ment Health. 2019;23(6):706–10. doi: 10.1080/13607863.2018.1450841 29553806

[34] Liu KPY, Chan CCH, Chu MML, Ng TYL, Chu LW, Hui FSL, et al. Activities of daily living performance in Dementia. Acta Neurol Scand. 2007;116(2):91–5. doi: 10.1111/j.1600-0404.2007.00800.x 17661793

[35] Mao H-F, Chang L-H, Tsai AY-J, Huang W-NW, Tang L-Y, Lee H-J, et al. Diagnostic accuracy of instrumental activities of daily living for dementia in community-dwelling older adults. Age Ageing. 2018;47(4):551–7. doi: 10.1093/ageing/afy021 29528375

[36] Katz S, Downs TD, Cash HR, Grotz RC. Progress in development of the index of ADL. Gerontologist. 1970;10(1):20–30. doi: 10.1093/geront/10.1_part_1.20 5420677

[37] Benjamini Y, Hochberg Y. Controlling the false discovery rate: a practical and powerful approach to multiple testing. J R Stat Soc Ser B Stat Method. 1995;57(1):289–300. doi: 10.1111/j.2517-6161.1995.tb02031.x

