# Supplementary material for: Randomized clinical trial of ICECaP (Individualized Coordination and Empowerment for Care Partners of Persons with Dementia): Primary mental health and burden outcomes
Source: PLoS One. 2025 Jan 24;20(1):e0309508. doi: 10.1371/journal.pone.0309508 (PMC11760562; doi:10.1371/journal.pone.0309508)
Supplement: S1 File — (PDF) [file pone.0309508.s001.pdf]

Contemporary Clinical Trials 137 (2024) 107418

Contents lists available at ScienceDirect

Contemporary Clinical Trials

journal homepage: www.elsevier.com/locate/conclintrial

# Individualized Coordination and Empowerment for Care Partners of Persons with Dementia (ICECaP): Study rationale and protocol

Virginia T. Gallagher [a,*], Shannon E. Reilly [a], George Worthington [b], James Patrie [c], Carol Manning [a]

[a] Department of Neurology, School of Medicine, University of Virginia, PO Box 801018, Charlottesville, Virginia 22908, USA
[b] Division for Community Living, Virginia Department for Aging and Rehabilitative Services, USA
[c] Department of Public Health Sciences, School of Medicine, University of Virginia, USA

ARTICLE INFO

*Keywords:*
Dementia
Care partner
Caregiver
Psychosocial intervention
Care coordination

ABSTRACT

The majority of care for >10 million older adults with dementia (PWD) in the United States depends on at least on 11 million unpaid care partners (CPs). CPs are at greater risk of adverse physical, psychological, and cognitive health outcomes relative to non-caregiving peers. The goal of this paper is to establish the rationale, design, and protocol for a pilot randomized control trial to test the efficacy of the CP-focused intervention, ICECaP: Individualized Coordination and Empowerment for Care Partners of Persons with Dementia. ICECaP involves the assignment of a trained dementia care coordinator to a CP. The care coordinator maintains at least monthly contact with the CP with hybrid delivery (in-person, phone, e-mail, and video calls) and provides individualized support with care coordination for the CP navigating the PWD's care in a complex healthcare system, as well as supportive counseling, psychoeducation, and skills training for the CP. This trial will compare outcomes from baseline to 12-months among CPs who receive ICECaP versus routine care (controls). Outcomes include CP depression, burden, anxiety, and quality of life; CPs' reactions to the behavioral symptoms of dementia; and use of support services for the PWD. This trial will also assess mechanisms of intervention efficacy including changes in CP dementia knowledge, caregiving preparedness, self-efficacy, and optimism. Publication of this intervention protocol will benefit other dementia care teams seeking to support CPs and PWDs.

## 1. Introduction

Over 10 million U.S. older adults experience cognitive decline due to suspected Alzheimer's disease (AD), and millions more experience cognitive and functional changes due to non-AD etiologies (i.e., cerebrovascular disease, Lewy Body disease, frontotemporal degeneration) [1]. Care for these individuals depends mostly on 11 million unpaid care partners (CPs) who provide >16 billion annual care hours valued at $272 billion [1]. CPs for persons with dementia (PWD) have increased morbidity, potentially higher mortality, poorer physical health, mental health, and cognitive health (including higher risk for dementia) compared to non-CPs [2–10]. Negative biopsychosocial effects of caregiving affect CPs' quality of life, undermine their capacity to provide care, and lead to poorer outcomes and increased institutional placement for PWDs, thereby increasing burden on public systems [11–15].

Psychosocial interventions for CPs of PWDs have been developed to reduce CP burden, stress, and depression; increase positive aspects of caring; improve knowledge/ability to provide care; and delay or even prevent institutionalization of PWDs [16]. These interventions tend to have small to medium effect sizes on the aforementioned outcomes [17]. Intervention types include psychoeducation, cognitive-behavioral therapy, skills training, case management, and care coordination; intervention structure includes individual and group formats and active versus passive engagement. Intervention format ranges from in-person to telehealth (phone, video, or combination), and mixed in-person and telehealth. Factors that tend to yield positive intervention outcomes include individualized support, active CP participation (versus passive information provision), therapeutic psychosocial support, and provision of knowledge and information about dementia and caregiving [16–18].

Care coordination, also referred to as care navigation, is an individualized approach to supporting CPs and helping them and their care recipients (PWD) overcome barriers to care, including navigating

* Corresponding author.
*E-mail addresses:* cdd3me@uvahealth.org (V.T. Gallagher), shannon.reilly@virginia.edu (S.E. Reilly), George.Worthington@dars.virginia.gov (G. Worthington), jpatrie@virginia.edu (J. Patrie), cm4r@virginia.edu (C. Manning).

https://doi.org/10.1016/j.cct.2023.107418
Received 19 September 2023; Received in revised form 20 November 2023; Accepted 17 December 2023
Available online 21 December 2023
1551-7144/© 2023 Elsevier Inc. All rights reserved.

Downloaded for Anonymous User (n/a) at UVA Health from ClinicalKey.com by Elsevier on August 08, 2024.
For personal use only. No other uses without permission. Copyright ©2024. Elsevier Inc. All rights reserved.

complex health systems, financial/insurance systems, and community programs [19,20]. Care coordination reduces the impact of health disparities in other clinical populations, such as cancer, and is showing promise in improving outcomes of PWDs [19,21]. Collaborative care coordination, which involves assistance with navigation of health systems, resource identification, and some degree of social/emotional support, has been piloted with CPs of PWDs by several organizations in the United States and has demonstrated improved quality of life for the PWD, reduced PWD emergency department visits, and reduced CP depression and burden [19,21,22]. In 2023, the Centers for Medicare & Medicaid Services announced the establishment of a new voluntary nationwide model – the Guiding an Improved Dementia Experience (GUIDE) Model – for supporting people living with dementia and their unpaid CPs [23]. GUIDE requires that the needs of CPs be assessed and addressed and that each PWD (and their associated CP) have a care navigator [23]. Therefore, it is essential that the scientific community publish and evaluate models of CP support and PWD/CP care coordination.

With support from our partners at the Administration on Aging, Administration for Community Living, the U.S. Department of Health and Human Services, the Jefferson Area Board for Aging, the Virginia Department for Aging and Rehabilitative Services, and the Department of Defense, our team at UVA Health developed an intervention that combines individualized elements of care coordination, supportive counseling, psychoeducation, and skills training and is delivered in a hybrid setting – combining an initial home visit, ongoing telehealth interactions via phone, email, and HIPAA-compliant video calls, and accompaniment to PWDs' clinic visits. A dementia care coordinator (referred to as care coordinator going forward) is assigned to a CP and makes at least monthly contact with the CP. This intervention, entitled Individualized Coordination and Empowerment for Care Partners of Persons with Dementia (ICECaP), is a person-centered intervention directed at the CP and is not a dyadic intervention. ICECaP was delivered to CPs of PWDs from 2018 to 2021 for purposes of feasibility and acceptability testing. Based on the initial feasibility testing among 35 CPs followed for 24 months, ICECaP was modified and refined to prepare for pilot efficacy testing described in this manuscript.

The goal of this paper is to establish the rationale, design, and protocol for a pilot randomized control trial to test the intervention efficacy of ICECaP, in preparation for refinement and optimization for scalability and dissemination. Specifically, CPs who participate in ICECaP for 12 months will be compared to those in an active control group (Control), who receive informal caregiving support via standard care appointments for the PWD in the multidisciplinary dementia care clinic, but not the individualized support of a care coordinator.

## 2. Methods

### 2.1. Theoretical approach

This intervention is grounded in an adapted CP stress and health theoretical framework (Fig. 1), which the authors developed by adapting and modifying past models, primarily utilizing the caregiving biopsychosocial stress model developed by Savla & Zarit (2015) [24], by integrating "three well-established paradigms —namely, Pearlin, Mullan, Semple, and Skaff's (1990) stress process model; Lazarus and Folkman's (1984) transactional stress, appraisal, and coping model; and Selye (1952) and McEwen's (1998) concepts of allostatic load." We added to this framework by explicitly integrating aspects of the stress process model depiction from Lee et al. (2021) [25] to more easily visualize identified targets for intervention. Finally, we emphasized social determinants of health in the care partner's context/background based on the National Institute on Aging Health Disparities Research Framework. Our integrated framework was generated to create a heuristic for physical and mental health outcomes among CPs of PWDs that includes social determinants of health and acknowledges the explicit relationship between physical and mental health.

ICECaP directly targets the "modifiable mediators" in the theoretical framework with the expectation that ≥monthly contact with the care coordinator will result in increased social and emotional support and yield improvements in individualized areas of need, such as psychoeducation on effectively addressing PWDs' behaviors, stress coping skills, dementia knowledge, and awareness of other resources. This will in turn result in improved CP mental health and quality of life. We will also evaluate the extent to which other social determinants of health and other baseline contextual factors in the CP may influence intervention efficacy .

ICECaP is also grounded in the Benjamin Rose Institute on Aging's Care Consultation™ Model (BRI-CCM), in combination with person-centered care practices. BRI-CCM is an evidence-based program designed to help professionals assist and support CPs of patients living with chronic conditions through assessment, care planning, and ongoing support. BRI-CCM emphasizes providing education to CPs and, to the extent it is helpful, PWDs, connecting them to community resources, and strengthening family and other social support networks. The BRI-CCM entails an initial assessment (ideally in-person) and follow-up phone, video-calls, and emails. During these communications, care coordinators use person-centered practices to collaborate with CPs and patients [26].

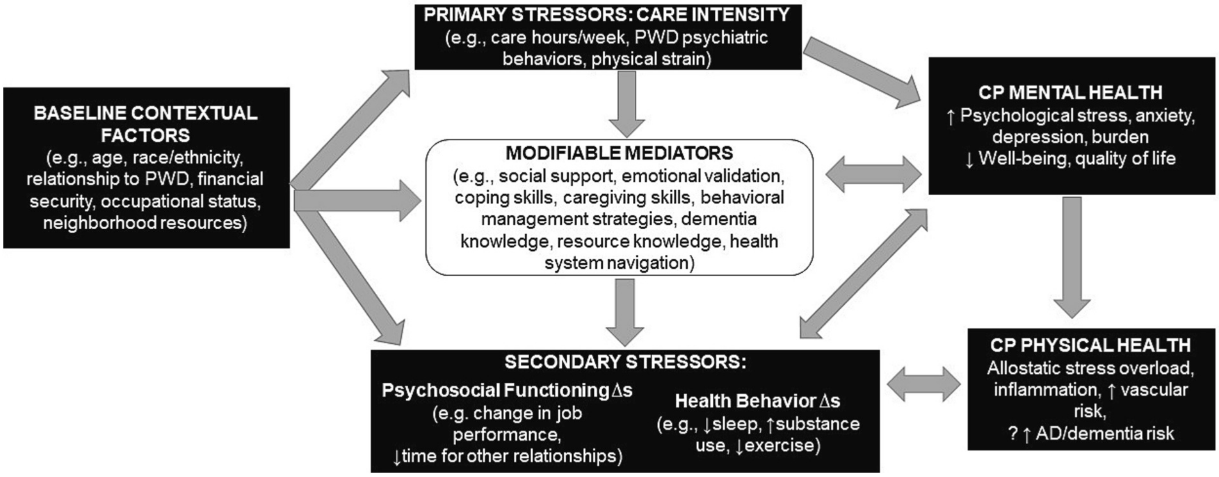


**Fig. 1.** Theoretical care partner (CP) stress framework.
PWD = person with dementia; AD = Alzheimer's disease.



Downloaded for Anonymous User (n/a) at UVA Health from ClinicalKey.com by Elsevier on August 08, 2024.
For personal use only. No other uses without permission. Copyright ©2024. Elsevier Inc. All rights reserved.

Researchers have investigated multiple facets of dementia caregiving using the BRI-CCM, including: engagement of PWD, quality of life for dementia care partners, and care coordination programming's impacts on decreased hospital admissions and emergency department visits [27–29]. While the BRI-CCM model has been applied and adapted for both individual (i.e., CP or patient) and dyadic (i.e., CP and patient) use [27–29], ICECaP is a CP-centered intervention targeted at improving outcomes among the CP.

### 2.2. Research aims

Aim 1: Does the intervention work? Determine whether ICECaP improves CP burden, symptoms of depression and anxiety, reactions to the PWD's behavioral symptoms of dementia, and CP quality of life.

**Hypothesis 1a**. After controlling for baseline characteristics, including level of PWD functional dependence, CPs in ICECaP will experience improvement from baseline to 12 months (post-intervention) on measures of CP burden, depression, anxiety, and quality of life, and their reactions to PWDs' behavioral symptoms, whereas Controls will not.

**Hypothesis 1b**. CPs in ICECaP will maintain their positive improvements in burden, depression, quality of life, and reactions to dementia behavioral symptoms at 18-month follow-up, after controlling for baseline PWD functional dependence.

Aim 2: Why does the intervention work? Evaluate the primary mechanisms of ICECaP intervention efficacy (effects on burden, depression, anxiety, quality of life, and reactions to PWD behaviors).

**Hypothesis 2a**. Within CPs who participated in ICECaP, positive effects in primary outcome measures will be correlated with pre- to post-intervention changes in dementia knowledge, caregiving preparedness, self-efficacy, and optimism.

**Hypothesis 2b**. CPs in ICECaP will experience greater improvements from baseline to 12-months in dementia knowledge, caregiving preparedness, self-efficacy, and optimism relative to Controls, after controlling for baseline level of PWD functional dependence.

*Exploratory aim 2c*: Within CPs in ICECaP, we will explore the extent to which changes on primary measures from pre- to post-intervention are predicted by baseline characteristics, specifically:

- *CP personal characteristics and social determinants of health*: CP age, race/ethnicity, education level, employment status, financial security
- *Caregiving context*: CP relationship to the PWD, co-dwelling status with the PWD, caregiving intensity (hours per week; numbers of basic and complex activities of daily living supported); caregiving duration (months); PWD quality of life, dementia severity at baseline, and number of neuropsychiatric symptoms at baseline.
- *Caregiving readiness:* baseline CP dementia knowledge, baseline CP preparedness for caregiving.

Understanding these exploratory associations will help inform future program optimization to best support those who need it most.

Aim 3: Does the intervention impact support service use for the PWD? Determine whether ICECaP increases CPs' use of ancillary support services for the PWD.

**Hypothesis 3**. CPs in ICECaP will report more hours of ancillary support services (i.e., home help/healthcare assistant, Meals on Wheels, adult daycare, care-related transportation) in the last 30 days at 12-month follow-up versus Controls, after controlling for baseline ancillary support service use and PWD functional dependence.

Exploratory Aim 4: How does engagement in and use of the intervention impact outcomes? Evaluate associations among intervention metrics and ICECaP CP outcomes.

*Aim 4a*: Among CPs in ICECaP, examine associations between CP intervention satisfaction scores and intervention metrics including frequency of CP-care coordinator contact, contact modality, and contact content.

*Aim 4b*: Examine associations between CP changes on primary outcome measures (Aim 1) and intervention metrics including frequency of CP-care coordinator contact, contact modality, and contact content.

### 2.3. Study design

#### 2.3.1. Participant recruitment and enrollment

CPs are recruited primarily from a Memory and Aging Care Clinic (MACC), a multidisciplinary clinic comprised of 2 neurologists specializing in neurodegenerative disorders, 2 geriatricians, 4 neuropsychologists, ≥ 4 neuropsychology postdoctoral fellows, one nurse practitioner, one pharmacist, one social worker, one nurse coordinator, one speech-language pathologist, and one occupational therapist. Potential CP participants who are likely to meet criteria in Table 1 are identified and referred to study staff by multidisciplinary team members during standard care appointments for the PWD in MACC. Potential CP participants are then formally screened by clinical research coordinators using inclusion criteria. All participants undergo full informed consent procedures prior to initiating any study activity.

#### 2.3.2. Randomization procedure

CPs are randomized to engage in 12 months of ICECaP or 12 months in the active control group using a random permuted block randomization scheme to ensure inter-study-arm assignment balance throughout enrollment (see Fig. 2). PROC plan procedure of SAS version 9.4 (SAS Institute Inc., Cary, NC) was used to generate the randomization. Fourteen blocks of ten assignments were generated: five ICECaP and five active control assignments, in random order. Due to the nature of the intervention, it cannot be masked once a participant has been randomized to a condition.

#### 2.3.3. Retention strategies

In the ICECaP group, we expect that monthly outreach from the care coordinator promotes retention, particularly because CPs receive the intervention's supportive services without cost. To reduce attrition in the active control group, control participants are offered the ICECaP intervention after 12 months of no support and completion of the 12-month questionnaires.

#### 2.3.4. Sample size and withdrawal

The target sample size is $n = 140$ CPs, 50% ICECaP and 50% active control, based on power analyses indicating that this is the required sample size to achieve 80% power for detecting a small to medium effect size ($d = 0.3$ to 0.5) at a significance level of $\alpha = 0.05$ when comparing baseline to 12-month change on Hypothesis I outcomes between ICECaP vs. Control groups.

CPs are withdrawn from the study if a) they do not respond to care coordinator contact for 2 months despite 3 attempts, b) the PWD moves out of the state (because of requirement to be followed by a member of

**Table 1**
Participant inclusion/exclusion criteria.

| Inclusion criteria | Exclusion criteria |
|---|---|
| Self-identified primary CP for community-dwelling PWD with clinician-diagnosed mild to moderate dementia | Associated PWD in nursing home |
| CP age ≥ 18 | CP age < 18 |
| CP has basic spoken and written English or Spanish skills | Associated PWD previously had >1 appointment in UVA MACC follow-up clinic |
| CP has internet access at home | |



Downloaded for Anonymous User (n/a) at UVA Health from ClinicalKey.com by Elsevier on August 08, 2024.
For personal use only. No other uses without permission. Copyright ©2024. Elsevier Inc. All rights reserved.

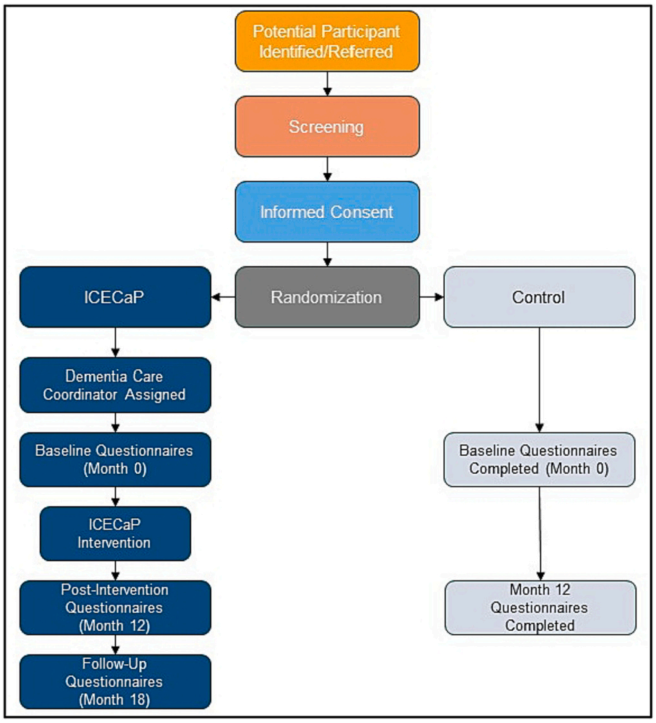


**Fig. 2.** Study design overview.

the healthcare team at the home institution), c) the PWD moves permanently to a residential facility (assisted living, memory care unit, or nursing home), or d) death of PWD or CP. If withdrawal is due to the PWD moving permanently to a residential facility or the death of the PWD, the study team reaches out to the CP 2 months following the event and requests completion of post-intervention questionnaires if CPs are willing.

#### 2.3.5. ICECaP intervention

*2.3.5.1. Care coordinator assignment and baseline questionnaires completion.* Within 7 days of informed consent and randomization into ICECaP, a care coordinator is assigned to the CP and baseline questionnaires are sent to the CP. Baseline questionnaires include a needs assessment to which the care coordinator can refer during the initial session. A clinical research coordinator prompts CPs to complete baseline questionnaires if needed.

*2.3.5.2. Initial contact.* Within 7 days of completion of study questionnaires, the care coordinator makes initial contact with the CP by email to introduce themselves and to schedule an initial session. They also email the CP the ICECaP Care Partner Guide, a 5-page document providing information about the intervention's purpose, care coordinator contact information, program limits (i.e., that it is not a crisis intervention service) and expectations, CP program requirements, and withdrawal procedures.

*2.3.5.3. Initial session.* The care coordinator and the CP schedule an initial session within 4 weeks of the initial contact (ideally within 2 weeks). The initial session may take place via phone, video, or at the CP's home if they live within 2 h of the care coordinator. A home visit is offered to all CPs who live within 2 h of the care coordinator unless contraindicated (e.g., CP or co-dweller has compromised immune system and does not wish to have visitors). The purpose of the initial session is to establish rapport and to discuss initial needs. Fig. 3 describes the process of the 12-month intervention, from the initial session to termination.

*2.3.5.4. Ongoing, ≥ monthly contact.* All contacts require a minimum of 15 min of care coordinator time. Each care coordinator attempts to have at least once monthly contact with the CP (no prescribed maximum), recognizing that some CPs may need substantially more support and interaction in any given month (e.g., during times of crisis or significant change for the PWD). CPs are also encouraged to reach out to their care coordinator for additional support as needed via email or phone. The flexibility in contact frequency per month is consistent with a person-centered approach to care coordination. Care coordinators are required to offer to attend PWD appointments in MACC, with permission from the CP, to aid continuity of care and recommendation follow-through. At our site, PWDs typically have 1–2 follow-up MACC appointments per year as part of standard care; these appointments are not a requirement for the ICECaP intervention. Care coordinators are required to check email and phone voicemail throughout the day to be responsive to CP needs.

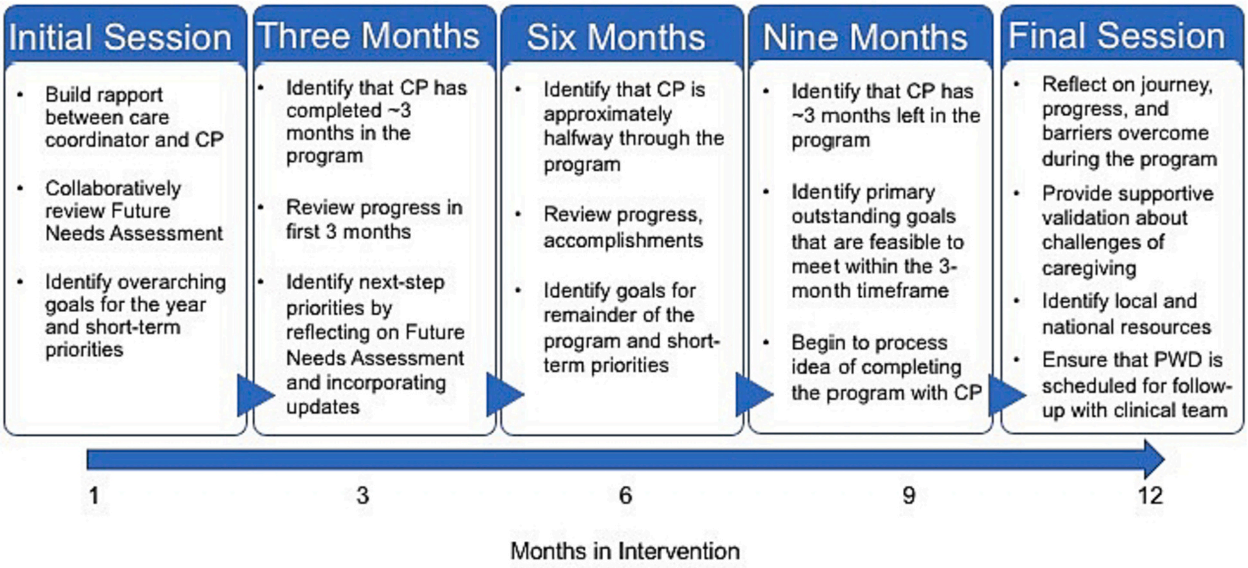


**Fig. 3.** ICECaP intervention process.



Downloaded for Anonymous User (n/a) at UVA Health from ClinicalKey.com by Elsevier on August 08, 2024.
For personal use only. No other uses without permission. Copyright ©2024. Elsevier Inc. All rights reserved.

*2.3.5.5. Modality/setting of contact.* Contact modalit(y)ies aredetermined by CP preference but can include email exchange, phone call, video (i.e., HIPAA-compliant Zoom), and/or in person in the clinic, at the CP's home, or in the community at an appropriate location (e.g., touring a residential facility).

*2.3.5.6. Contact content.* The content of CP-care coordinator contact is CP-centered and driven by the specific needs of the CP. Care coordinators are responsible for providing supportive counseling and a diverse array of services, specific to the needs of each CP, which often include: education on dementia, information about and referrals to community-based organizations, behavioral symptom management training, and assistance with determining eligibility for and enrolling in available community and government-sponsored programs. Please see Table 2 for categorization and examples of contact content. After each contact, the care coordinator sends a follow-up email containing relevant notes and resources. After each contact, the care coordinator sends a follow-up email containing relevant notes and resources.

As an example, a contact might involve the CP and care coordinator discussing new behavioral and psychological symptoms of dementia observed by the CP. The care coordinator might assist with identifying situational triggers for the PWD, discussing possible behavioral management strategies, identifying a behavioral management strategy preferred by the CP, discussing implementation of the strategy, and the care coordinator may consult with healthcare team members to identify whether an underlying medical condition (e.g., urinary tract infection) may need possible assessment. Care coordinators provide an individualized level of support for any given recommendation. As another example, if after an initial session at the CP/PWD's home the care coordinator identifies that a grab bar would reduce safety concerns, the care coordinator may solely recommend grab bar installation and offer suggestions about where the CP can purchase a grab bar. Based on individualized CP needs, additional support and guidance the care coordinator may provide include: reaching out to the PWD's nurse practitioner to further assess need for the grab bar (if the CP is reluctant), collaboratively reviewing options for grab bar purchase and installation with the CP (if the CP feels overwhelmed with choices), identifying grants to help pay for the grab bar (if there is financial hardship), and/or arranging for a home-health occupational therapy visit (if the CP is unsure how to guide the PWD to use the grab bar once installed).

**Table 2**
Contact content.

| | Content Coding Category | Example(s) |
|---|---|---|
| A | Behavioral management strategies not related to safety | Managing sundowning behaviors, visual hallucinations; attention redirection techniques |
| B | Safety strategies that are not behavior management | Prompting purchasing of medical alert device, automatic stove shut-off, grab-bars for shower |
| C | Strategies for both behavior management AND safety | Strategies for discouraging PWD driving |
| D | Strategies for activities of daily living | Prompting purchase of smart pillboxes, establishing alarms to eat |
| E | Administration/case management | Scheduling medical visits, consulting with other providers, coordinating respite stays |
| F | Referrals to other providers | Referral to speech therapy |
| G | Education about navigating diagnosis and disease progression | Psychoeducation regarding diagnosis and prognosis |
| H | Guidance and education on community resources | Directing CP to local area agency on aging, Meals on Wheels |
| I | Guidance and education on financial resources or benefits | Discussing Medicare/Medicaid benefits; discussing available respite care vouchers |
| J | Guidance and support for long-term care planning | Options counseling for assisted living versus in-home care |
| K | Providing emotional support | Supportive counseling for changing relationship dynamics, discussing anticipatory grief |
| M | Guidance and education about research/clinical trials | Providing information about brain donation, local clinical trials |
| N | Guidance and education about legal resources | Assistance with updating power of attorney or advance directives |
| O | Rapport-building | Establishing familiarity, warmth, and trust between the CP and care coordinator above and beyond usual empathic care |
| P | Lifestyle, quality of life, and enrichment/engagement resources | Specific enrichment classes at a local senior center for the CP and/or PWD |

*2.3.5.7. Documentation of contacts.* Contact date, modality, and duration, and session content coding are documented in a database for study purposes and in the electronic health record to facilitate coordination and continuity of care among the PWD's providers. Direct care coordinator-CP communication time is recorded in minutes (minimum = 15 min). Indirect care coordinator time (i.e., activities not involving CP-care coordinator direct contact, such as researching resources, travel time, coordination with other providers, documentation in the research database and electronic health record) is also recorded in minutes.

### 2.4. Care coordinator background, training, and supervision

#### 2.4.1. Qualifications

A bachelor's degree is strongly preferred for care coordinators. A background in social work, counseling, case management, mental health, and/or dementia is strongly emphasized. While no prior local experience is necessary, in the development phase of this intervention it became clear that duration of experience in the relevant local community is associated with increased knowledge and awareness of community resources and increased familiarity with healthcare providers. As such, it is helpful to have more experienced care coordinators work with less experienced care coordinators or care coordinators who are new to the region. In the intervention development phase of ICECaP, characteristics of successful care coordinators included strong communication skills (for use both with the CP and the healthcare team), cognitive flexibility, ability to adapt to needs and questions that arise in real-time, creativity with adapting strategies to the context of the CP, self-awareness of emotional boundaries and triggers, resiliency in the face of frequent sad content, internal motivation (as positive feedback from CPs does not occur frequently due to feelings of CPs being overwhelmed), ability to simultaneously validate CP's experience and gently confront CP to effect positive change, and willingness to engage in hybrid work environments (i.e., travel outside of the clinic to CPs' homes).

#### 2.4.2. ICECaP intervention training

Prior to study-specific training, care coordinators complete online Collaborative Institutional Training Initiative (CITI), HIPAA, and Benjamin Rose Institute on Aging's Care Consultation™ Model (BRI-CCM) trainings. For ICECaP intervention training, care coordinators complete a protocol of online training and resources from various agencies (see Supplementary Material), with explicit content for ensuring sound understanding of dementia processes, disease progression, and common caregiving challenges. Training includes options counseling (i.e., interactive decision support process) training and introduction to and relationship-building with local resource leaders. Care coordinators are also required to review extensive resource materials that were gathered for the ICECaP intervention. Completion of online trainings and review of resource materials are documented in a training log. Care coordinators are instructed that a primary goal of the program is to empower CP self-efficacy such that if the program is "successful," the CP will feel like they have strategies and resources (or awareness of available resources) to support the PWD.



Downloaded for Anonymous User (n/a) at UVA Health from ClinicalKey.com by Elsevier on August 08, 2024.
For personal use only. No other uses without permission. Copyright ©2024. Elsevier Inc. All rights reserved.

#### 2.4.3. Ongoing supervision

Care coordinators meet as a group once weekly with the principal investigator (PI) and co-investigator (co-I), who are both neuropsychologists with expertise in psychological intervention and neurodegenerative diseases. Care coordinators meet twice monthly with the ICECaP team, which includes the PI, co-I, care coordinators, clinical research coordinator(s), and the data management team to discuss intervention logistics, troubleshoot challenges, and complete documentation (e.g., reporting for funding mechanisms).

#### 2.4.4. Caseload management

For full-time care coordinators whose time is 100% allocated to delivery of the ICECaP intervention, 50 CPs per care coordinator are expected. For this study, we anticipate 3 care coordinators will serve 70 CPs (approximately 23 CPs per care coordinator), keeping in mind that at our site, care coordinators' time is not 100% allocated to delivery of this intervention. At least 2 care coordinators are required due to the need for peer consultation, peer emotional support, and peer resource sharing.

### 2.5. Intervention fidelity

To ensure training fidelity, all training logs are reviewed by the PI prior to a care coordinator initiating contact with CPs. To ensure intervention fidelity, summary and documentation of care coordinators' $\geq$ monthly contact per CP is reviewed at each meeting.

To ensure session content topics are documented consistently in the database, care coordinators are required to review all session content codings with the PI/co-I and other care coordinators in regular group meetings after the first 2 contacts with a new participant. Care coordinators are encouraged to discuss contact content codings that do not clearly fit into a single coding type or content that appears to fit into multiple content codings with the PI/co-I and other care coordinators in weekly group meetings. The group then decides on the best fitting coding for the content.

### 2.6. Outcomes

See Table 3.

All data are stored using REDCap [42] electronic data capture tools hosted by the University of Virginia and monitored monthly by the clinical research coordinator for missing data.

### 2.7. Analytic plan

#### 2.7.1. Analytic overview

This study includes four unique but unified analytical Aims. Aim 1 is examines whether ICECaP improves target CP outcomes. Aim 2 evaluates the primary mechanisms of ICECap intervention efficacy. Aim 3 assesses whether the ICECaP intervention impacts support service use for the PWD. Aim 4 explores how engagement and use of the ICECaP intervention impact outcomes and evaluates potential associations among intervention metrics and ICECaP CP outcomes. Detailed descriptions of Aim 1, Aim 2, Aim 3, and Aim 4 analytical plans are provided in the Supplementary Materials. Between-group comparisons will involve covariance adjustment for PWD functional dependence at baseline.

## 3. Results

IRB approval for this study was granted on 9/8/2020. Funding for this study is provided by Department of Defense (AZ190036). Recruitment began on 3/1/2021 and data collection is estimated to be completed by 3/14/2025.

## 4. Discussion

This paper describes the rationale, design, and protocol for ICECaP, an individualized intervention for CPs of PWD that involves at least monthly contact between the CP and a trained care coordinator via both in-person and telehealth modalities. We developed this intervention to specifically target CP burden, depression, and quality of life (among other factors) by using one-on-one at least monthly contacts between the CP and the care coordinator. Based on our theoretical framework (see Fig. 1), we expect that the intervention will improve these outcomes by way of improving CP knowledge about dementia, preparedness for caregiving, self-efficacy, and optimism.

While data collection has already begun for this iteration of the intervention, the scientific review process has highlighted important opportunities for future refinement and improvement of ICECaP. Specifically, future applications of the intervention should include more specific and frequently reviewed benchmarks for intervention fidelity.

**Table 3**
Study measures.

| Aim | Construct | Self-report Measure | Measure Details |
|---|---|---|---|
| *Primary Outcome Measures:* Evaluated Baseline vs. 12-month, ICECaP vs. Control | | | |
| Aim1 | CP Burden | Zarit Burden Interview (ZBI)* [30] | 22-item measure assessing degree of CP burden |
| Aim1 | CP Depression | Center for Epidemiologic Studies Depression Scale—Revised (CESD-R)* [31] | 20-item measure assessing symptoms of depression |
| Aim 1 | CP Reaction to Behavioral Symptoms | Revised Memory and Behavior Problem Checklist (RMBPC)* [32] | 24-item measure assessing CP-reported problematic behaviors in PWD and CP's reaction |
| Aim 1 | CP Quality of Life | WHO (Five) Well-Being Index (WHO-5)* [33] | 5-item measure assessing dimensions of psychological well-being |
| Aim 1 | CP Anxiety | Geriatric Anxiety Inventory (GAI) [34] | 20-item measure assessing symptoms of anxiety |
| Aim 2 | CP Dementia Knowledge | Dementia Knowledge Assessment Tool Version 2 (DKAT2) [35] | 21-item measure assessing CP's foundational-level knowledge of dementia (e.g., dementia progression, support, and care) |
| Aim 2 | CP Caregiving Preparedness | Preparedness for Caregiving Scale (PCS) [36] | 8-item measure that assesses CPs' feelings of preparedness for multiple domains of caregiving |
| Aim 2 | CP Self-Efficacy | General Self-Efficacy scale (GSE) [37] | 10-item measure assessing feelings of self-efficacy |
| Aim 2 | CP Optimism/Pessimism | Life-Orientation Test-Revised (LOT-R) [38] | 10-item measure assessing CP's attitudes about the future |
| Aim 2 | CP-reported PWD Basic ADLs | Katz Index of Independence in Activities of Daily Living [39] | 6-item measures assessing independence in basic daily activities |
| Aim 2/ Covariate | CP-reported PWD Complex ADLs | Lawton Instrumental Activities of Daily Living Scale [40] | 8-item measure assessing independence in complex daily activities |
| Aim 3 | CP-reported Ancillary Resource Utilization | Resource Utilization in Dementia (RUD), questionnaire version 4.0 [41] | Standard tool assessing resource utilization among patients with dementia |
| Aim 4 | CP Intervention Satisfaction | – | 15-item measures assessing satisfaction with the intervention; administered to ICECaP only |

All measures were completed by ICECaP CPs and Controls at baseline and 12-months; *denotes measures completed at 18-month follow-up by ICECaP CPs only.



Downloaded for Anonymous User (n/a) at UVA Health from ClinicalKey.com by Elsevier on August 08, 2024.
For personal use only. No other uses without permission. Copyright ©2024. Elsevier Inc. All rights reserved.

Additionally, depending on attrition in this study, more intensive retention strategies may be needed in the future.

This pilot study will determine the efficacy of the intervention and explore the mechanisms of efficacy. It will also investigate how social determinants of health and caregiving contextual factors influence positive effects on primary outcome measures. In addition, we will explore the extent to which various intervention engagement metrics (e.g., contact frequency, modality, and content) influence degree of positive outcomes. Collectively, this study will lead to future optimization trials that will ultimately inform scalability and dissemination of this psychosocial CP intervention.

## Funding

This work was funded by the Department of Defense (AZ190036); program development and pilot feasibility testing was funded by the Virginia Department for Aging and Rehabilitative Services (45918). REDCap support at the University of Virginia is supported in part by the National Center for Advancing Translational Sciences of the National Institutes of Health under Award # UL1TR003015.

## CRediT authorship contribution statement

**Virginia T. Gallagher:** Conceptualization, Methodology, Writing – original draft. **Shannon E. Reilly:** Data curation, Investigation, Methodology, Project administration, Supervision, Writing – review & editing. **George Worthington:** Conceptualization, Investigation, Project administration, Software, Supervision, Writing – review & editing. **James Patrie:** Data curation, Formal analysis, Writing – original draft. **Carol Manning:** Conceptualization, Data curation, Funding acquisition, Investigation, Methodology, Project administration, Resources, Supervision, Writing – original draft, Writing – review & editing.

## Declaration of Competing Interest

The authors declare that they have no known competing financial interests or personal relationships that could have appeared to influence the work reported in this paper.

## Data availability

No data was used for the research described in the article.

## Acknowledgements

We wish to acknowledge the fantastic team that contributed to the development and delivery of the ICECaP intervention, including Anna Arp, Elizabeth Boyd, Samantha Fields, Jessica Samet, Scott Sperling, and Yenifer Valera.

## Appendix A. Supplementary data

Supplementary data to this article can be found online at https://doi.org/10.1016/j.cct.2023.107418.

## References

[1] 2022 Alzheimer''s disease facts and figures, Alzheimers Dement. 18 (4) (2022 Apr) 700–789 (PMID: 35289055).
[2] K.B. Dassel, D.C. Carr, P. Vitaliano, Does caring for a spouse with dementia accelerate cognitive decline? Findings from the health and retirement study, Gerontologist 57 (2) (2017 Apr 1) 319–328.
[3] A.N. Leggett, A.J. Sonnega, M.C. Lohman, Till death do us part: intersecting health and spousal dementia caregiving on caregiver mortality, J. Aging Health 32 (7–8) (2020) 871–879 (PMCID: PMC7187632).
[4] B. Lovell, M.A. Wetherell, The cost of caregiving: endocrine and immune implications in elderly and non elderly caregivers, Neurosci. Biobehav. Rev. 35 (6) (2011 May) 1342–1352 (PMID: 21333674).
[5] R. von Känel, J.E. Dimsdale, P.J. Mills, S. Ancoli-Israel, T.L. Patterson, B. T. Mausbach, I. Grant, Effect of Alzheimer caregiving stress and age on frailty markers interleukin-6, C-reactive protein, and D-dimer, J. Gerontol. A Biol. Sci. Med. Sci. 61 (9) (2006 Sep) 963–969 (PMID: 16960028).
[6] M. Pinquart, S. Sörensen, Differences between Caregivers and Noncaregivers in Psychological Health and Physical Health: A meta-Analysis. Psychology and Aging vol. 18, American Psychological Association, US, 2003, pp. 250–267.
[7] P.P. Vitaliano, J. Zhang, J.M. Scanlan, Is caregiving hazardous to one's physical health? A meta-analysis, Psychol Bull. 129 (6) (2003 Nov) 946–972 (PMID: 14599289).
[8] M. Perkins, V.J. Howard, V.G. Wadley, M. Crowe, M.M. Safford, W.E. Haley, G. Howard, D.L. Roth, Caregiving strain and all-cause mortality: evidence from the REGARDS study, J. Gerontol. B Psychol. Sci. Soc. Sci. 68 (4) (2013 Jul) 504–512 (PMCID: PMC3674731).
[9] D.L. Roth, L. Fredman, W.E. Haley, Informal caregiving and its impact on health: a reappraisal from population-based studies, Gerontologist 55 (2) (2015 Apr) 309–319. PMCID: PMC6584119.
[10] B.D. Capistrant, J.R. Moon, L.F. Berkman, M.M. Glymour, Current and long-term spousal caregiving and onset of cardiovascular disease, J. Epidemiol. Commun. Health. 66 (10) (2012 Oct 1) 951–956. BMJ Publishing Group Ltd. PMID: 22080816.
[11] C.Y. Chiao, H.S. Wu, C.Y. Hsiao, Caregiver burden for informal caregivers of patients with dementia: a systematic review, Int. Nurs. Rev. 62 (3) (2015 Sep) 340–350 (PMID: 26058542).
[12] D.M. Bass, K.S. Judge, A.L. Snow, N.L. Wilson, R. Morgan, W.J. Looman, C. A. McCarthy, K. Maslow, J.A. Moye, R. Randazzo, M. Garcia-Maldonado, R. Elbein, G. Odenheimer, M.E. Kunik, Caregiver outcomes of partners in dementia care: effect of a care coordination program for veterans with dementia and their family members and friends, J. Am. Geriatr. Soc. 61 (8) (2013 Aug) 1377–1386 (PMID: 23869899).
[13] D.M. Bass, K.S. Judge, A.L. Snow, N.L. Wilson, W.J. Looman, C. McCarthy, R. Morgan, C. Ablorh-Odjidja, M.E. Kunik, Negative caregiving effects among caregivers of veterans with dementia, Am. J. Geriatr. Psychiatry 20 (3) (2012 Mar) 239–247 (PMID: 22251867).
[14] M.E. de Vugt, F.R.J. Verhey, The impact of early dementia diagnosis and intervention on informal caregivers, Prog. Neurobiol. 110 (2013 Nov) 54–62 (PMID: 23689068).
[15] J.E. Gaugler, F. Yu, K. Krichbaum, J.F. Wyman, Predictors of nursing home admission for persons with dementia, Med. Care 47 (2) (2009 Feb) 191–198 (PMID: 19169120).
[16] C. Dickinson, J. Dow, G. Gibson, L. Hayes, S. Robalino, L. Robinson, Psychosocial intervention for carers of people with dementia: what components are most effective and when? A systematic review of systematic reviews, Int. Psychogeriatr. 29 (1) (2017 Jan) 31–43 (PMID: 27666669).
[17] E. Walter, M. Pinquart, How effective are dementia caregiver interventions? An updated comprehensive meta-analysis, Gerontologist. 60 (8) (2020 Nov 23) 609–619 (PMID: 33226434).
[18] J. Hopwood, N. Walker, L. McDonagh, G. Rait, K. Walters, S. Iliffe, J. Ross, N. Davies, Internet-based interventions aimed at supporting family caregivers of people with dementia: systematic review, J. Med. Internet Res. 20 (6) (2018 Jun 12), e9548.
[19] A. Bernstein, K.L. Harrison, S. Dulaney, J. Merrilees, A. Bowhay, J. Heunis, J. Choi, J.E. Feuer, A.M. Clark, W. Chiong, K. Lee, T.L. Braley, S.J. Bonasera, C. Ritchie, D. Dohan, B.L. Miller, K.L. Possin, The role of care navigators working with people with dementia and their caregivers, J. Alzheimers Dis. 71 (1) (2019) 45–55 (PMCID: PMC7004209).
[20] K.B. Hirschman, M. McHugh, B. Morgan, An integrative review of measures of transitions and care coordination for persons living with dementia and their caregivers, Alzheimers Dement (N Y) 9 (3) (2023) e12391. PMCID: PMC10404587.
[21] K.L. Possin, J.J. Merrilees, S. Dulaney, S.J. Bonasera, W. Chiong, K. Lee, S. M. Hooper, I.E. Allen, T. Braley, A. Bernstein, T.D. Rosa, K. Harrison, H. Begert-Hellings, J. Kornak, J.G. Kahn, G. Naasan, S. Lanata, A.M. Clark, A. Chodos, R. Gearhart, C. Ritchie, B.L. Miller, Effect of collaborative dementia care via telephone and internet on quality of life, caregiver well-being, and health care use: the care ecosystem randomized clinical trial, JAMA Intern. Med. 179 (12) (2019 Dec 1) 1658–1667 (PMCID: PMC6777227).
[22] B.A. Kallmyer, D. Bass, M. Baumgart, C.M. Callahan, S. Dulaney, L.C. Evertson, S. Fazio, K.S. Judge, Q. Samus, Dementia care navigation: building toward a common definition, key principles, and outcomes, Alzheimer's & Dementia: Translational Research & Clinical Interventions. 9 (3) (2023), e12408.
[23] CMS.gov, Biden-Harris Administration Announces Medicare Dementia Care Model, Available from: https://www.cms.gov/newsroom/press-releases/biden-harris-administration-announces-medicare-dementia-care-model, 2023 Jul 31.
[24] S.H. Zarit, J. Savla, Caregivers and stress, in: G. Fink (Ed.), Stress: Concepts, Cognition, Emotion, and Behavior [Internet], Academic Press, San Diego, 2016, pp. 339–344 [cited 2023 Jun 13]. Available from: https://www.sciencedirect.com/science/article/pii/B978012800951200042X.
[25] K. Lee, M. Yefimova, F. Puga, C.E. Pickering, Gender differences in caregiver burden among family caregivers of persons with dementia, Journal of Gerontological Nursing. SLACK Incorporated 47 (7) (2021 Jul) 33–42.
[26] M.J. Koren, Person-centered care for nursing home residents: the culture-change movement, Health Affairs (Project Hope). 29 (2) (2010).
[27] L. Darlak, D.M. Bass, K.S. Judge, N. Wilson, W. Looman, C. Mccarthy, R. Morgan, K. Maslow, M.E. Kunik, Engagement of veterans with dementia in partners in dementia care: an evidence-based care coordination program, Journal of Applied Gerontology 36 (5) (2017) 570–591 (Los Angeles, CA: SAGE Publications.



Downloaded for Anonymous User (n/a) at UVA Health from ClinicalKey.com by Elsevier on August 08, 2024.
For personal use only. No other uses without permission. Copyright ©2024. Elsevier Inc. All rights reserved.

[28] H. Moon, A.L. Townsend, C.J. Whitlatch, P. Dilworth-Anderson, Quality of life for dementia caregiving dyads: effects of incongruent perceptions of everyday care and values, The Gerontologist. Oxford University Press 57 (4) (2017) 657–666.
[29] D.M. Bass, K.S. Judge, K. Maslow, N.L. Wilson, R.O. Morgan, C.A. Mccarthy, W. J. Looman, A.L. Snow, M.E. Kunik, Impact of the care coordination program "Partners in Dementia Care" on veterans' hospital admissions and emergency department visits, Alzheimer's & Dementia: Translational Research & Clinical Interventions 1 (1) (2015) 13–22. Elsevier Inc.
[30] M. Bédard, D.W. Molloy, L. Squire, S. Dubois, J.A. Lever, M. O'Donnell, The Zarit burden interview: a new short version and screening version, Gerontologist. 41 (5) (2001 Oct) 652–657 (PMID: 11574710).
[31] W.W. Eaton, C. Smith, M. Ybarra, C. Muntaner, A. Tien, Center for Epidemiologic Studies Depression Scale: Review and Revision (CESD and CESD-R). The Use of Psychological Testing for Treatment Planning and Outcomes Assessment: Instruments for Adults 3rd ed., Vol. 3, Lawrence Erlbaum Associates Publishers, Mahwah, NJ, US, 2004, pp. 363–377.
[32] L. Teri, P. Truax, R. Logsdon, J. Uomoto, S. Zarit, P.P. Vitaliano, Assessment of behavioral problems in dementia: the revised memory and behavior problems checklist, Psychol. Aging 7 (4) (1992 Dec) 622–631 (PMID: 1466831).
[33] C.W. Topp, S.D. Østergaard, S. Søndergaard, P. Bech, The WHO-5 well-being Index: a systematic review of the literature, Psychother. Psychosom. 84 (3) (2015) 167–176 (PMID: 25831962).
[34] C. Johnco, A. Knight, D. Tadic, V.M. Wuthrich, Psychometric properties of the geriatric anxiety inventory (GAI) and its short-form (GAI-SF) in a clinical and non-clinical sample of older adults, Int. Psychogeriatr. 27 (7) (2015 Jul) 1089–1097 (PMCID: PMC4501012).
[35] C. Toye, L. Lester, A. Popescu, F. McInerney, S. Andrews, A.L. Robinson, Dementia knowledge assessment tool version two: development of a tool to inform preparation for care planning and delivery in families and care staff, Dementia (London). 13 (2) (2014 Mar 1) 248–256 (PMID: 24339059).
[36] P.G. Archbold, B.J. Stewart, M.R. Greenlick, T. Harvath, Mutuality and preparedness as predictors of caregiver role strain, Res. Nurs. Health 13 (6) (1990 Dec) 375–384 (PMID: 2270302).
[37] R. Schwarzer, M. Jerusalem, Generalized self-efficacy scale, in: J. Weinman, S. Wright, M. Johnston (Eds.), Measures in Health Psychology: A User's Portfolio, NFER-NELSON, Windsor, 1995, pp. 35–37.
[38] M.F. Scheier, C.S. Carver, M.W. Bridges, Distinguishing optimism from neuroticism (and trait anxiety, self-mastery, and self-esteem): A reevaluation of the life orientation test, in: Journal of Personality and Social Psychology vol. 67(6), American Psychological Association, US, 1994, pp. 1063–1078.
[39] S. Katz, A.B. Ford, R.W. Moskowitz, B.A. Jackson, M.W. Jaffe, Studies of illness in the AGED. The index of ADL: a standardized measure of biological and psychosocial function, JAMA. 185 (1963 Sep 21) 914–919 (PMID: 14044222).
[40] M.P. Lawton, E.M. Brody, Assessment of older people: self-maintaining and instrumental activities of daily living, Gerontologist. 9 (3) (1969) 179–186 (PMID: 5349366).
[41] A. Wimo, A. Gustavsson, L. Jönsson, B. Winblad, M.A. Hsu, B. Gannon, Application of resource utilization in dementia (RUD) instrument in a global setting, Alzheimers Dement. 9 (4) (2013 Jul) (429–435.e17. PMID: 23142433).
[42] P.A. Harris, R. Taylor, B.L. Minor, V. Elliott, M. Fernandez, L. O'Neal, L. Mc Leod, G. Delacqua, F. Delacqua, J. Kirby, S.N. Duda, REDCap Consortium, The REDCap consortium: building an international community of software platform partners, J. Biomed. Inform. 95 (2019 Jul), 103208 (PMCID: PMC7254481).



Downloaded for Anonymous User (n/a) at UVA Health from ClinicalKey.com by Elsevier on August 08, 2024.
For personal use only. No other uses without permission. Copyright ©2024. Elsevier Inc. All rights reserved.
