# Supplementary material for: Randomized clinical trial of ICECaP (Individualized Coordination and Empowerment for Care Partners of Persons with Dementia): Primary mental health and burden outcomes
Source: PLoS One. 2025 Jan 24;20(1):e0309508. doi: 10.1371/journal.pone.0309508 (PMC11760562; doi:10.1371/journal.pone.0309508)
Supplement: S2 File — (PDF) [file pone.0309508.s002.pdf]

Future Needs Assessment

Please select topics that you would like further information/resources on from the list below. Your answers will provide your Dementia Care Manager (DCM) with insight into areas that you may need additional assistance.

---

1) Todays Date

____

---

2) Please select topics that you would like further information/resources on from the list below. Your answers will provide your Clinical Care Coordinator (CCC) with insight into areas that you may need additional assistance. Select all that apply.

- ☐ Understanding your diagnosis of memory or thinking problems
- ☐ Telling others about the diagnosis
- ☐ Maintaining healthy relationships with family and friends
- ☐ Emotional wellbeing
- ☐ Memory or thinking problem related behaviors
- ☐ Changes with language/speech and communication strategies
- ☐ Daily strategies
- ☐ Legal and financial planning
- ☐ Elder abuse and exploitation
- ☐ Falls, home safety, disaster management
- ☐ Wandering
- ☐ Driving
- ☐ Medications
- ☐ Sleep
- ☐ Exercise
- ☐ Nutrition
- ☐ Care partner self-care, supports, and resources
- ☐ Task management - online applications (apps) or resources
- ☐ Social engagement - online community of newly diagnosed individuals, community centers
- ☐ Clinical trials, trial match (Alzheimer's Association)
- ☐ Social programs, such as Memory Café and Arts Fusion (Fusion First and Fusion Plus programs) from Alzheimer's Association

---

3) If there are any other topics, or if there are topics that you consider a priority, please list them here.

____
